# Evaluate the outcome of pneumonectomy for stage III a-N2 NSCLC

**DOI:** 10.1186/1749-8090-10-S1-A206

**Published:** 2015-12-16

**Authors:** Sungjoon Park, Hyo-Jun Jang, Eunjue Yi, Sukki Cho, Sanghoon Jheon, Kwhanmien Kim

**Affiliations:** 1 Department of Thoracic and Cardiovascular Surgery, Seoul National University Bundang Hospital, Seoul National University College of Medicine, 166 Gumi-ro, Bundang-gu, Seongnam-si, Gyeonggi-do 463-707, Seoul, Korea

## Background/Introduction

The effect of surgery in patients with III a-N2 non-small cell lung cancer is not certain.

## Aims/Objectives

We intend to evaluate the outcome of pneumonectomy in III a-N2 non-small cell lung cancer.

## Method

We retrospectively reviewed the cases of patients with III a-N2 non-small cell lung cancer, who underwent pneumonectomy from April 2003 to January 2014 at a single institution.

## Results

During the study, 184 patients underwent surgical resection of stage III a-N2 non-small cell lung cancer. Among them, 35 patients had pneumonectomy, 29 patients (83%) were male, and 6 (17%) were female. The median age was 57 years (43 yrs-77 yrs) old, and the median follow up duration was 77 month. 27 patients (77%) had induction chemotherapy before operation. Right pneumonectomy was performed in 13 patients (37%), and Left pneumonectomy was done in 22 patients (63%). The 5-year survival was 58% in pneumonectomy, 28% in the right pneumonectomy and 76% in the left pneumonectomy, respectively. Left pneumonectomy(p = 0.021) and complete resectability (p = 0.017) were positive prognostic factor, but the induction chemotherapy before operation was negative prognostic factor. (p = 0.040)

## Discussion/Conclusion

The side of operation and complete resectability are positive prognostic factor, but the induction chemotherapy is negative prognostic factor for pneumonectomy in III a-N2 NSCLC patients.

